# Supplementary figures and images for: Cytokines and Chemokines Involved in Hepatitis B Surface Antigen Loss in Human Immunodeficiency Virus/Hepatitis B Virus Coinfected Patients
Source: J Clin Med. 2021 Feb 18;10(4):833. doi: 10.3390/jcm10040833 (PMC7922731; doi:10.3390/jcm10040833)

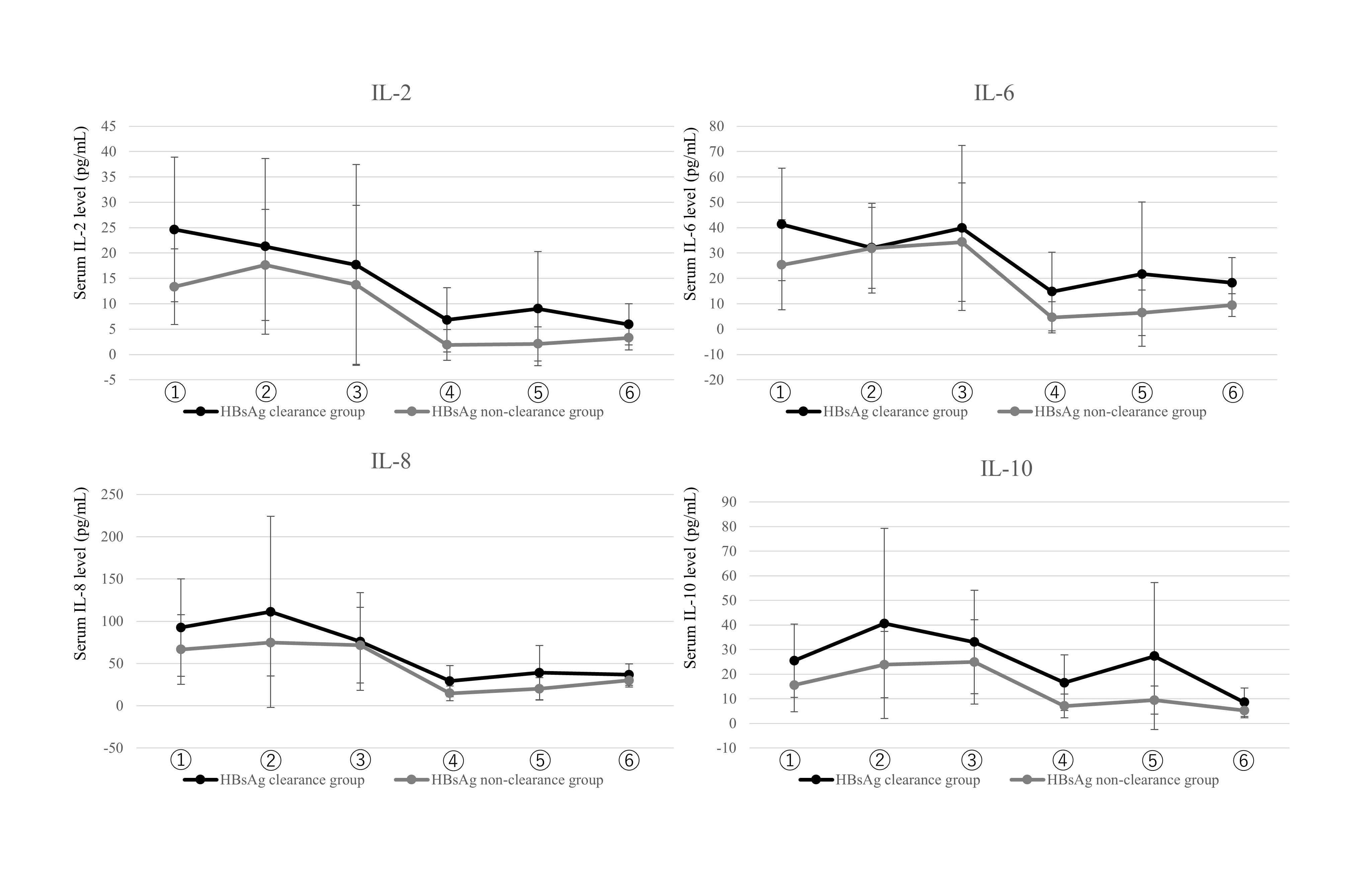

Supplement: Supplementary file 1 [file jcm-10-00833-s001.zip › Figure S1.JPG]

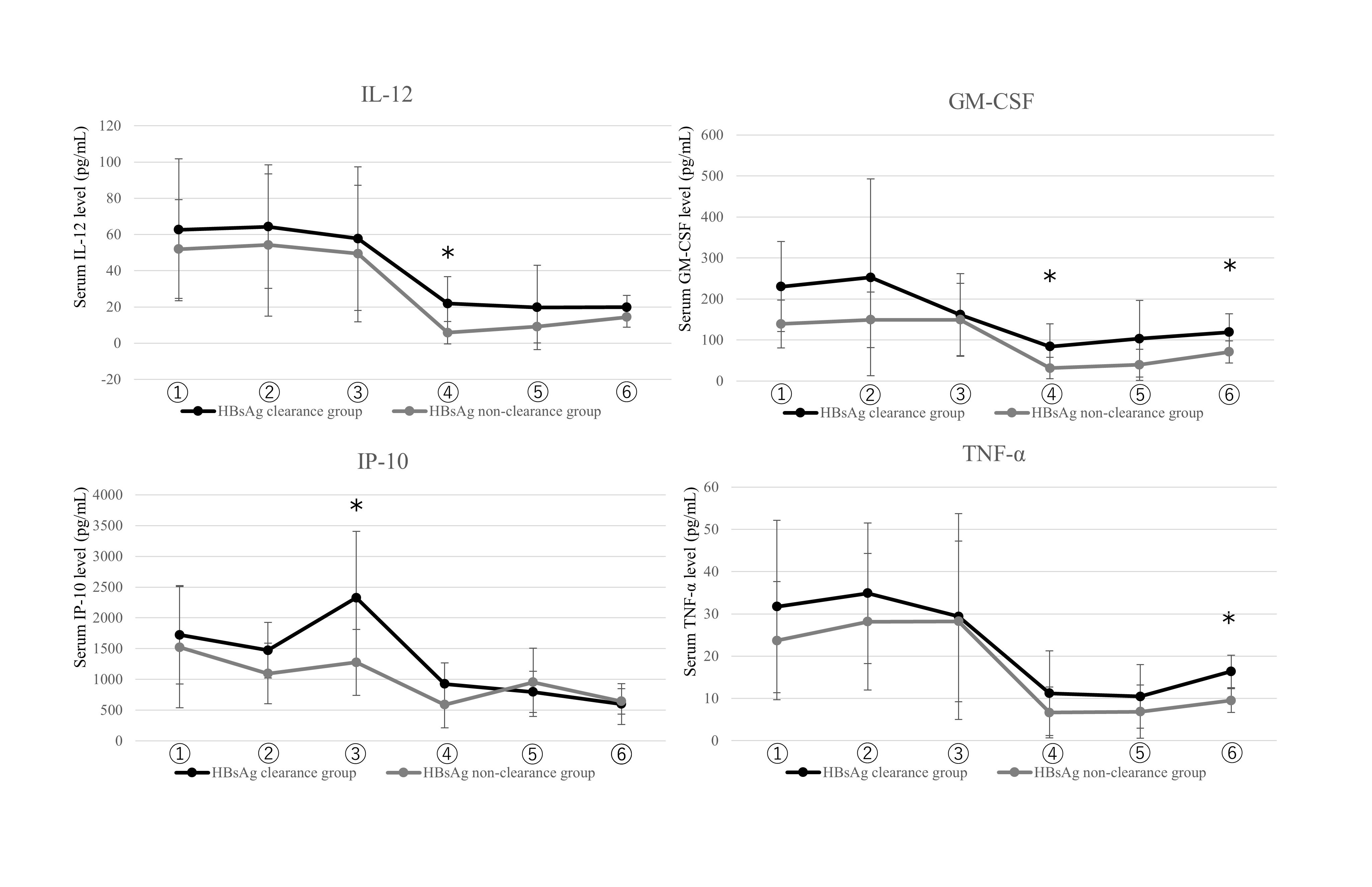

Supplement: Supplementary file 1 [file jcm-10-00833-s001.zip › Figure S2.JPG]
